# Book Reviews

**Published:** 1833-01

**Authors:** 


### CRITICAL ANALYSES.

Quæ laudanda forent, et quæ culpanda, vicissim,

Illa prius, cretâ; mox hæc, carbone, *notamus.*—PERSIUS.

*Medico-Chirurgical Transactions.* Vol. XVII.—8vo. pp. 527; four plates. Longman and Co., London.

THE rapidity of communication which now exists, not only from place to place, but from mind to mind, favors that morbid irritability which is getting more and more notorious, to publish cases, essays, and memoirs, on the spur of the moment; for no sooner has a case been witnessed, or an idea conceived, than it is ushered "into this breathing world, scarce half made up," in some one or other of our weekly, daily, and almost hourly periodicals. Time was, when a treatise might be esteemed the fruit of experience, and a book the labour of a life; when consideration was bestowed on opinions before they were promulgated; while now, if they are considered at all, they must be considered after publication, and not seldom, we opine, give their authors, who have printed in a hurry, just cause to repent at leisure. Such is the precocious constitution of the post-haste periodicals of the present day, that, with breathless ardour, sometimes print the beginning of a speech before its conclusion has passed the speaker's lips; and the commencement is thus read by gaping multitudes before the termination has been heard by the listening few: hence, we have sometimes wondered that any materials have been spared at all for the pages of the more staid and slowly-moving monthlies and quarterlies, and especially for those still more tardigradous literary planets whose revolutions occupy an entire year. Yet we do find that these meteors of the day have not entirely consumed the materials which used to be devoted to the support of the more steady burning lights which so long have illumined the sphere of science; and the present volume of the Medico-Chirurgical Transactions affords abundant proof that, if trifles are sent to trifling channels, the more important are reserved for the nobler streams.

The contributors to the present volume are Drs HODGKIN, HALL, and LEE; Messrs. LAWRENCE, COOPER, LANGSTAFF, BARLOW, HAWKINS, WOOD, HEWLETT, BRODIE, TRAVERS, HOWSHIP, SHAW, and EVANS; who have furnished sixteen memoirs, all of them on interesting, and several on very important topics. It would be impossible, within the space which we dedicate to reviews, to analyse the whole of these papers; we shall, therefore, direct our attention chiefly to those which will suffer least by abridgment, and which we conceive are of the most practical importance, contenting ourselves with a reference to the others, which our readers will find well worthy their perusal.

The volume opens with some *Observations on Tumors, with Cases*, by W. LAWRENCE, Esq., F.R.S., President of the Society; in which, after some good general remarks, and an attempt to restrict the meaning of the term "tumor," as defined by Mr. ABERNETHY, to new productions, or, rather, to "accidental productions, either similar to the normal tissues, or dissimilar, developed in the cellular or adipous structures, or in the substance of any organ," which "productions are often unattended with any increased magnitude, so that the etymology of the word must be disregarded," the author contends (and we think successfully) against the commonly received opinions that "either the effusion of blood, and its coagulation, and the subsequent organization of the coagulum," or "the effusion and organization of coagulating lymph," or "chronic inflammation," are, one or other, the causes of these morbid states. He says,

"When various explanations are given of any living process, we shall find that its nature is not understood. There can be only one true account of the matter, and when that has been discovered, the others are discarded. It seems to me in the present instance, that neither of the explanations above mentioned offers a satisfactory solution of the phenomena.

"According to either of these views, tumors ought to pass through successive stages, and to present different appearances at different periods of their development. For instance, we ought to find them at first as masses of coagulated blood, or coagulating lymph, and then to observe various degrees of transition from those substances to the textures which characterise the perfect growth. Observation, however, discloses nothing of this kind: tumors, in their earliest state and smallest size, have their peculiar structure as well marked as in their subsequent progress and full development. An adipous tumor, not exceeding the bulk of a pea, differs only in size from one as large as the head. Effusions of blood into the cellular texture from external violence are of daily occurrence: hardly an individual escapes them. If such extravasations could become organised and then form tumors, the latter should prevail almost universally. We see, however, that the blood, thus poured out, either disappears by absorption, or irritates the surrounding parts, and causes suppuration, by which it is expelled. No instance has yet been adduced in which such an effusion has been converted into a tumor." (P. 6.)

The author confesses that he has "nothing to offer in place of the explanations to which he has now objected. It seems to him that the circumstances which determine the production of tumors generally, or of any particular kind of growth, are entirely unknown to us; nor ought we to feel surprised at our ignorance respecting these aberrations of nutrition, when we are quite in the dark as to the mode in which the process is accomplished in its natural or normal state; when we know nothing of the differences in arrangement or operation which lead to the varied results of vascular action; when we are unable to explain how the capillary vessels of one part deposit muscle, of another bone, of a third fat; how one gland secretes bile, another urine, and a third saliva." (P. 9.)

Among the cases here recorded, that of the "*Large Cellular Tumor occupying the Labium Pudendi and Buttock*" is the most rare and the most important; we shall therefore extract the essential parts.

"I was consulted, in the year 1826, by a lady twenty-eight years of age, handsome, very well formed, of fair complexion and light hair. She came into my room with her sister, looking very strong and healthy, and said that she wished to have my opinion on a rupture. She turned aside to loosen her dress, that I might examine the part, and was longer than I expected in making the necessary arrangement. I looked round, expecting to meet with a hernial tumor in the groin or bend of the thigh, perhaps as large as a walnut or an egg, when, to my utter astonishment, I saw hanging from one of the buttocks a mass about twice the size of my head, of which the accompanying drawings exhibit a posterior and a lateral view. It was greater in breadth than the tranverse measurement of the two thighs, which, in a tall and very well made person like this lady, were of good size. The complaint had existed for four years, and had not grown fast during the first two. It had given no pain, and even at present was only troublesome by its weight, bulk, and inconvenient position. It interrupted no function, and did not even impair any; indeed, the general appearance of the patient was that of perfect health and strength. It had commenced at the posterior part of the left labium pudendi, and had extended gradually along the buttock and behind the os coccygis. The tumor was a soft, but not fluctuating mass, and slightly subdivided into large lobes. The skin was loosely connected to it, so that it could be pinched up into large folds: it had become partially excoriated from pressure and friction, and was consequently rather red and rough on the prominent parts of the swelling; but in other respects it was healthy. Some veins of moderate size could be obscurely seen on the surface. The basis was the smallest portion of the tumor, which expanded below into a pendulous mass, rather broader than the two thighs. The basis reached from the coccyx to the left labium, and from the edge of the gluteus magnus to the anus. The greatest circumference of the swelling was thirty-two inches; it was twenty-one inches round at the basis, eleven inches from the latter to the middle of its inferior edge, and eight in the line of the basis from the coccyx towards the trochanter.

"The basis of the tumor was quite moveable on the subjacent parts, but it was of uncertain extent towards the front; that is, I could not determine how far it reached inwards beyond the labium, or towards the cavity of the peritoneum. The patient said that her medical attendant had thought it was a rupture, and I therefore examined carefully whether any impulse was produced in it on coughing, or whether the swelling was continued along the side of the vagina. These points were not clear, but the absence of the symptoms which a protrusion large enough to form such a swelling must have caused, sufficiently disproved the notion of rupture. The propriety of taking another opinion having been suggested, this lady consulted Mr. Wardrop, and we determined that the disease ought to be removed, as we could discover no connexion with any internal part, although we could not trace the boundary of the growth satisfactorily towards the labium and vagina.

"I removed the tumor on the 9th of September, 1826, making two incisions, one on the outer, and the other on the inner side, and detaching integument enough to cover the denuded surface after the operation. The skin was easily separated, and the base of the tumor was readily detached from the parts beneath, its situation being quite superficial, so that merely a few fibres of the gluteus magnus were exposed. But the anterior portion, which advanced into the labium, could not be eradicated; it passed inwards along the side of the vagina, on which there was an evident drag, when this part was pulled: I therefore cut it through. The part thus divided was tough, with a compact fibrous structure, and a light reddish-grey colour. There was free bleeding from numerous vessels of various sizes, which I did not stop to tie, and a large quantity of blood was lost, when the patient became very faint, and the hemorrhage ceased, so there was no necessity for ligatures. The edges of the incision were brought together by eight sutures, and the wound was thus as completely closed as the thickened state of the skin detached from the tumor would admit, when the patient was put to bed in a very faint state, in which she continued till night. She, however, spoke and looked very well, so that I could entertain no apprehension about her, although the pulse at the wrist was barely perceptible. Two grains of crude opium at bedtime." (P. 11.)

Some slight constitutional disturbance followed the removal of the tumor, which, however, was easily subdued, and on the 23d the patient left town; the wound being then nearly closed.

"The tumor consisted of a fleshy mass with a somewhat elastic feel, approaching nearly to fluctuation: its substance undulated and trembled when pressed or moved. On cutting through it, the texture was found nearly uniform throughout, being rather more compact on the prominent part, where it must have suffered pressure in sitting and lying, and have partaken of the irritation which had caused excoriation of the surface. Its tint was reddish grey. Its structure was tough and fibrous, and consisted of condensed cellular tissue entirely free from fat. It was left during the night in a large dish, and in the morning a very considerable quantity of fluid had escaped from it. This was not subjected to chemical analysis; it is therefore merely a conjecture that it may have been similar to the fluid frequently found in the interstices of the natural cellular tissue.

"This lady, who had regained her strength rapidly after leaving London, and had continued in perfect health, paid me a visit on the 13th of March, 1827, when I examined the cicatrix and found the part quite sound. The skin, which had formed, on its separation from the tumor, large thick folds, had shrunk considerably, so that the natural outline of the buttock was nearly restored. The entrance of the vagina was natural, and nothing like return of swelling could be discovered on introducing the finger. Soon afterwards she married, and I did not see her again till the spring of 1828, when she was far advanced in pregnancy, and had experienced a considerable reproduction of the tumor. It distended the loose skin left from the operation, filled the left labium at its back part, and could be felt through the vagina as a kind of cord ascending towards the pubes. She went through her confinement very well, aud I removed the swelling again on the 1st of August, 1828. The anterior prolongation was carefully traced on this occasion: it ascended along the left side of the vagina, becoming gradually smaller, and seemed to pass under or behind the arch of the pubes. As I was putting it on the stretch to trace it to its termination, it suddenly gave way, and I found that it had tapered quite to a point, the end appearing entire, as if the whole morbid growth had come away. The part removed was equal to about one third of the mass formerly extirpated, which it exactly resembled in structure. The edges were brought together by sutures, and the wound was covered with cold damp cloths. The healing went on rapidly, and the patient was able to return to the country on the eighth day. There has been no reproduction of the swelling."* (P. 15.)

The second paper contains the report of a very interesting case, and it is so short that it will not need much abridgment.

*Some Account of a Case in which the Left Femur and the Fifth Rib on the Right Side were fractured in consequence of Disease, the Bladder being at the same time in the state of Carcinomatous Ulceration; with Observations.* By SAMUEL COOPER, Professor of Surgery in the University of London, &c.

"On the 10th of November, 1831, I was desired by Mr. Blair, a surgeon in Great Russell street, Bloomsbury, to visit C. Askey, aged sixty-three, a gentleman's coachman, in Montague mews, Russell square, who had been for several months afflicted with severe pain in the left hip, pelvis, and loins; continual uneasiness in the glans penis; and frequent inclination to make water, which came away with difficulty, and sometimes blended with a considerable quantity of blood.

"About half an hour before I saw the patient, a loud snap was suddenly heard by his wife and daughter in one of his limbs, as he was turning himself in bed, and he was immediately seized with excruciating pain in the left thigh. On taking hold of the limb, I distinctly felt a crepitus not far below the trochanters; the limb was retracted; and it was manifest that the broken part of the bone was surrounded by a swelling, characterized by a remarkable degree of hardness.

"Long sufferings, and frequent returns of profuse hemorrhage from the bladder, had already reduced the patient to extreme emaciation and debility. The pulse was 130, irregular, and feeble; and the appetite entirely gone. Under these circumstances, conjoined, as they plainly were, with some organic disease of the bladder or kidneys, I applied splints to the broken femur, without any expectation of amendment of the health or cure of the fracture. That the bleeding and disturbance of the urinary organs were not produced by a calculus in the bladder was certain, because the quantity of blood in the urine was too copious to proceed from such a cause: the hemorrhage and pain had never been increased by exercise, nor even by the employment of driving a carriage; and, excepting at periods when blood came away, the urine was perfectly clear. The sound had also been introduced into the bladder several times, without detecting any calculus in it. After I had

* "In the course of the last year, a tumor of considerable size, exactly similar in structure to the foregoing, was removed from the left labium pudendi of a female, between twenty and thirty years of age, in St. Bartholomew's Hospital, by my colleague, Mr. Earle. This growth had formed and increased slowly, and without pain. It was loosely connected to the surrounding parts, except at its upper end, where it adhered more firmly by a kind of tough fibrous prolongation, which was cut through in order to detach the mass."

attended the patient a few days, the bleeding ceased, and the urine became natural and transparent; but a profuse hemorrhage commenced again on Sunday, November 20th, and he expired at ten o'clock the same night.

"On opening the abdomen, a particularly hard mass presented itself on the fundus of the bladder, directly behind the os pubis; so hard, that the gentleman who first examined it with his fingers supposed it to be a calculus. This indurated substance was found to constitute the base of a considerable fungus, situated upon the inner surface of this part of the bladder, and exceeding a crown piece in diameter. It was from this fungus that the copious bleedings had taken place from time to time, during the last five months of the patient's life.

"When the chest was examined, the fifth rib on the right side was found to have given way near its central part, the ends of the broken portion being at the same time involved in a scirrhous substance, which had quite as indurated a feel as the hardest kind of cartilage.

"The femur was surrounded at its broken part, and also for some extent above and below the fracture, with a mass of the same kind of morbid tissue already specified, which here formed a thick protuberance, increasing very much the deformity noticed about the hip directly after the occurrence of the fracture. The consistence of this tumor was rather softer towards the bone than more superficially, and from that part of it which lay near the fracture a reddish-brown thick fluid oozed out. The greater portion of it, however, was peculiarly firm, and thickly interspersed with innumerable fragments of osseous matter.

"As the patient had never complained of pain in the situation of the diseased and fractured rib, the discovery of such mischief was completely unexpected.

"The bladder contained about six ounces of turbid brown urine, and nearly as much coagulated blood.

"All the absorbent glands in the neighbourhood of the abdominal aorta were enlarged, (some of them to the size of a pigeon's egg,) and converted into the same kind of scirrhous substance observed around the fractured bones. * * *

"Some differences of opinion may exist about what name ought to be given to the tumor connected with the femur. The disease could not be regarded as fungus hæmatodes or medullary sarcoma, the hardness of the morbid tissue being too great, and the general intermixture of osseous matter with it constituting, perhaps, another difference. Besides, if it had been of this nature, the disease of the bladder would doubtless have been of a corresponding character; but this was not the case, for, though the ulcerated, fungous state of the inside of the fundus of that organ was of long standing, it did not form a large projecting mass, as happened in some other well-authenticated examples of fungus hæmatodes of the bladder, and especially in that published by my friend, Mr. Langstaff, in the eighth volume of the Transactions of this Society, p. 279. Neither were the fungous granulations connected with any soft, pulpy, medullary mass, but situated upon a particularly hard substance, which constituted their base. According to my views, this affection of the bladder must be deemed cancerous; and, if this inference be correct, may we not conclude that the tumor which grew round the femur, and led to its fracture, was of a similar nature: for it can hardly be doubted that the various forms of disease in this patient had a common origin, from some peculiarity of constitution with which they were essentially united? The fact of the absorbent glands in front of the spine, from the pelvis to the diaphragm, being all in a completely scirrhous state, seems to corroborate these observations.

"The indurated mass surrounding the broken femur was not continued from the medullary texture, nor in any way directly connected with it: yet this texture, when a longitudinal section was made of the bone, was found altered from its natural appearance, even as high up as the cancelli of the head of the femur, and also to the extent of two inches below the fracture. Throughout all this track, it was much paler than the healthy continuation of it lower down, and filled with pus. The boundary line between the healthy and diseased portions of the medullary texture is still completely manifest in the preparations now in my possession."

Mr. Lawrence's "*History and Dissection of a Case in which there had been Dislocation of the Ankle, with Fracture of the Fibula*;" Mr. Langstaff's "*Case of Polypus of the Uterus;*" and Dr. Hodgkin's Essay "*on some Morbid Appearances of the Absorbent Glands and Spleen*," we shall leave for private perusal, and make our next extract from Mr. Barlow's "*Case of Fractured Spine*," as it affords an illustration of an important point of practice.

"A man, twenty-eight years of age, whilst wheeling a barrow filled with gravel, was knocked down by the sudden falling of a tree, which some men were hewing; it struck him on the back, and fractured his spine. He was immediately conveyed to Writtle workhouse, where I visited him soon after his admission, being the surgeon of the establishment.

"I found him quite insensible, and he appeared almost lifeless. On examining the course of the spine, I discovered an angular projection in the situation of the last dorsal and first lumbar vertebræ.

"In a short time his powers rallied, but he was still faint, and his legs were completely paralysed. A small quantity of brandy and water was given.

"It seemed evident that the fractured parts were compressing the medulla spinalis, which induced me to attempt to place them in a more favorable position, by employing gradual extension. This was performed by persons pulling at the superior and inferior extremities, which had the desired effect; for the angular projection was greatly lessened, and the patient did not seem to experience any pain during the extension. He was placed horizontally on a firm mattress.

"The day after the accident, he complained of much pain, and of a burning sensation in the situation of the injured part. Leeches were applied, and afterwards an evaporating lotion with opium, which afforded great relief. The bowels and vesica not having been relieved from the time of his admission into the work-house, the urine was drawn off, and a brisk purgative given.

"What appeared very singular in this case was, the absence of febrile action, and of the symptoms denoting inflammation of the theca vertebralis; yet the lower part of the body was paralysed, and deprived of sensation. The urine was drawn off twice a day; his bowels were obstinately costive, and required active purgatives, such as croton oil, to relieve them.

"In this miserable state he continued about eight months, when sensation began to appear in his thighs and legs, and, with the assistance of a rope fixed to the upper part of the bedstead, he managed to raise himself in the half-bent position, without exciting the least sensation of pain in the spine.

"Hopes were at this time entertained that the case would terminate favorably, notwithstanding the serious injury which the spine had sustained.

"Although the sense of feeling in his inferior extremities improved, there still continued a want of power in the abdominal muscles and bladder. There was great difficulty in keeping his bowels open; but he would relieve the bladder by pressing with his hands firmly on the hypogastric region.

"After twelve months' confinement in bed, he was able to bear the fatigue of being carried down stairs, and of being drawn about in a chair in the open air.

"A few months from this period, he was attacked with a violent cold accompanied by fever, and was again confined to his bed; the integuments covering the left heel became inflamed, and sphacelation commenced. The febrile symptoms, however, subsided, and the integumental parts which had been destroyed by sloughing, were gradually repaired. But, although he recovered from this attack, his health began to decline; pus was voided with the urine, his bodily powers gradually diminished, and the integuments covering the sacrum became gangrenous, although great care had been taken to prevent such an occurrence.

"For fifteen days previous to his death, he had suffered from incessant vomiting, and his stomach scarcely retained sufficient nutriment to support life.

"*Dissection.* The injured portion of the spine having been removed, and the soft parts dissected off, a longitudinal section of the vertebræ and intervertebral substances was made, in order to show the changes which had been effected by the accident.

"The theca vertebralis was thickened, and of a darkish-red colour: the medulla spinalis was greatly softened, and diminished in size near the fractured part of the spine. The first lumbar vertebra had been fractured in a transverse direction, extending obliquely downwards and forwards through the upper third of its body. The upper part of the vertebral column, together with the upper fragment of the fractured vertebra, had been thrown forwards, the superior fragment resting on the fore and upper part of the inferior fragment, to which it was connected by callus. The corresponding articular process of the last dorsal and first lumbar vertebræ had been dislocated; those of the former having been thrown upwards and forwards. On the right side, the inferior articular process of the last dorsal vertebra was separated from the corresponding processes of the first lumbar, to the extent of the third of an inch; while their surfaces were rounded and nearly obliterated, in consequence of the length of time the patient had survived the accident. On the left side, the processes were about the same distance from each other, but connected by an intermediate portion of new bone. The foramen between the roots of the spinous processes was more than twice its natural dimensions. The ligamentum subflavum must have been ruptured.

"The most important point in the change which was produced by the accident consisted in the altered dimensions of the vertebral canal: immediately behind the line of fracture, it was diminished one half in calibre, and it is probable that the medulla spinalis projected in some degree through the enlarged opening between the spinous processes.

"The infundibula and pelves of the kidneys were dilated with urine mixed with pus; their internal surfaces were inflamed, as was the substance of the kidneys. There was a considerable quantity of urine and pus in the bladder, and its coats were thickened.

"According to Sir Astley Cooper's observations, patients with fractured lumbar vertebræ die within a month or six weeks; but he knew of one patient who lived two years, and then died of gangrene of the nates.

"In Mr. Samuel Cooper's Dictionary of Practical Surgery, in the description of fractures of the vertebræ, it is stated that any attempt to set fractures of the bodies of the vertebræ, even where they are known to exist, would be both useless and dangerous. In the case which I have described, it is evident that deviating from the general maxim did not produce any bad effects.

(To be continued.) p.132.

*A Treatise on Inflammations; explaining their Pathology, Causes, Consequences, and Treatment, with their Effects on the various Textures of the Body. Being an Extension of a "Dissertation on Inflammation of the Membranes," to which the Jacksonian Prize, for the year* 1828, *was awarded by the Royal College of Surgeons in London.* By GEORGE ROGERSON, Surgeon, of Liverpool. Vol. I.—8vo. pp. 459. Longman and Co., London.

THE Jacksonian prize having been awarded by the London College of Surgeons to Mr. ROGERSON, for his "Dissertation on Inflammation of the Membranes," he has judged it improper to permit his successful essay to stand alone the memorial of his authorship, and therefore, the statue being already wrought, he now seeks a pedestal on which it may be raised to a more commanding height than if left among the herd of treatises that issue daily from the press, and to which an individuality seldom is conceded, how distinct soever they each may be.

Hence has sprung this "extension" of an especial essay into a general "Treatise on Inflammations," of which the first volume, containing the "principles of inflammatory disorders and diseases," is now before us; and the second, to be devoted to the "pathology, causes, consequences, and treatment of the inflammations of the different textures of the body," is promised to be forthcoming shortly.

We rather expect that the second volume, from its table of contents, which accompanies the present, will be more to our liking than this first: for, however necessary a "history of opinions on the nature of inflammation," traced from the earliest epoch of our art to the present day, the "general principles of medicine," &c., may be to the author's design of beginning with the beginning, and raising his treatise from the foundation, still it is anything but interesting to the general reader, who has read the same matter over and over again, and often repeated in scarcely changing words. But, letting our distaste for these preliminary formalities pass, we readily declare that the introduction is written in a vivid style, and as much appearance of novelty as possible is given to the oft-told tale.

Mr. Rogerson is absolutely intolerant of ancient theories, the "*muscœ volitantes* of fruitful imaginations," not considering that such systems, however devious their course might be, still led the student up the steeps of science by a winding path, when infant philosophy was too weak to breast the hill, which now, even in maturity, all find it difficult to climb.

Our author very vigorously attacks the abstract *nature* of HIPPOCRATES, the *vis vitæ*, and the *Archæus;* but, while demolishing these Dagons of medical idolatry, he seems to us to be raising another golden calf in Bethel or in Dan. The fiery spirit of inflammation is said to be the sole source of all our ills; and, as cold is defined by philosophers to be the absence of heat, so here it would almost seem that health should only be regarded as the absence of disease.

We quote the opening chapter.

"Inflammation, affecting one or more of the various textures of the complicated machine of life, so changes their healthy structures as to become the parent of all our maladies, and frequently 'brings death into the world.' To its ravages, which proceed with more or less celerity on those important viscera whose natural organization is necessary to the preservation of existence, the great majority of mankind fall a deadly prey; while the remainder of our race, equally its victims, are conducted to the tomb, by quick or slow, but certain steps, through its blasting destruction of those parts which are of minor importance to vitality. Of all 'the numerous ills that flesh is heir to,' it is the first cause; and having once begun its baneful actions on the structures of the body, and established them, without being molested in its course, it proceeds, guided by fixed laws, from bad to worse, sometimes suddenly and violently tearing up the foundations of health, and sometimes gradually and silently sapping them, till it lays the noble edifice of life in ruins. It is also the cause of those more numerous minor ills, which, if they do not destroy life, embitter its hours, by giving occasional pangs, disturbing the general serenity of man's days, exciting petty irritations, or creating slight disorders of some duration. Pathology establishes these truths, and shews the origin of disease to be inflammation alone, by exhibiting its destructive work, and tracing its progress and effects on the various textures of which living bodies are composed. Morbid anatomists, then, are the painters of its deeds, and their descriptions present only so many pictures of its effects.

"Examine all the local changes of a diseased part, and trace their stages! The first stage will present a greater or less deranged state of the blood, capillaries, absorbents, nerves, and peculiar matter of the affected texture, with disordered actions of their powers and functions. This is inflammation. Sooner or later another stage succeeds, with violence or mildness, exhibiting striking alterations in the secretion, formation, or nutrition of the part, or its destruction. These are so many consequences or diseases of inflammation. The constitution is associated with these local changes by means of the systems. This is the fever of inflammation.

"Indeed, in all the different varieties and stages of diseased changes, observation of the natural processes will detect inflammation under some form or other: and, if tested by the spirit of experimental philosophy, all the diseased states under which texture and the constitution have ever existed, may be produced by inflammation artificially excited. Science, then, must approve of such an application of her laws, and acknowledge the simplicity of system founded upon it." (P. 1.)

Such is the design of this treatise, and such the basis on which the "general principles of medicine" here advocated stand. The whole of these principles we confess that we do not understand, and the correctness of several we think should rather have been proved than dogmatically assumed: take, for example, the two first.

"1. Matter is generally said to be of two kinds, living and dead; it is probable, however, that all masses of matter are only different modifications of each other's atoms, which consequently possess the same general properties; and there is no matter without power, nor power without matter.

"2. Living matter possesses sensation and motion." (P. 39.)

Does *all* living matter possess both sensation and motion? That the ancients often erred in considering diseases idiopathic which modern research has shewn to be rather symptomatic of local disorders, we willingly allow; but before these topical sources, these "*fontes et origines malorum,*" were discovered, we think they erred on the safe side; erred far less than many modern pathologists have done, who would make the brain, the lungs, or the bowels, the sole original seats of fevers, which our forefathers, not knowing of any especial organs to which they could be attributed, considered general or constitutional in their primary states, and named them idiopathic. Hence we must contend that the "16th principle," although it *may* be true, has not yet had its truth established; and from the 20th we entirely dissent.

"16. There is no fever without some local malady, and fevers are only the attendants of maladies, being, in fact, only symptoms of the disorder or disease of some part. They are the general or systemic symptoms of maladies; and as the local ones are the signs of physiological deviations immediately arising from the affected part, fevers are general symptoms or signs of physiological deviations, arising from the systems of the body sympathizing (according to the usual phrase) with the local affection." (P. 43.)

"20. All those remedies, therefore, which convert physiology into pathology, cannot be admitted; nor should any medicine be administered intended to produce or prolong a state of pathology. Strong counter-irritants, blisters, cauteries, issues, revulsives, excessive stimulants, which excite organized parts beyond the healthy standard, are improper medicines; so likewise are diaphoretics, diuretics, purgatives, emetics, and others, given in such a manner as to sweat, urinate, purge, vomit, &c., because these effects are the products of a state of structure and power different from that of health, creating, in short, disease.

"It is at war with common sense, as well as with observation, to think it possible to obtain physiology by changing a part into pathology. The above remedies, then, and indeed all kinds, should only be administered so as to procure a healthy state of power and matter: thus remedies from the class of purgative medicines should not be given in such a manner as to keep up a purging, for this would be creating a malady called diarrhœa, or an inflammation of the intestinal mucous membrane; but their effects should be to unload the alvine canal, to solicit proper secretions, and to restore a physiological state of structure, which will be manifested by healthy regular evacuations both in number, quantity, and quality." (P. 45.)

Against the 21st, which avers that "disorder of structure is frequently curable," we have no objection to make.

There is something startling in the hypothesis that the blood possesses an inherent power of self-motion: that it actually, not figuratively, *runs* through the blood-vessels, and does not need to be driven by a *vis a tergo*, or any other *vis*, the presence and power of which physiologists so long have laboured to explain.

"There is also a power of motion inherent in the blood, by which it is enabled to perform the circuit of the body, assisted by the propulsive action of the heart and vessels; and, divesting ourselves of all prejudices about the self-motion of a fluid, it will, I am convinced, be found the most rational mode of explaining the phenomena of the circulating fluid. This doctrine of the self-motion of the blood may not, from the difficulty of obtaining an unexceptionable proof, be supported by a direct experiment; but I trust it will be abundantly borne out by indirect evidence. I am persuaded that the powers of the vascular textures, singly or combined, will be found altogether inadequate to carry on the circulation; and this ought to convince even infidelity itself, that some other power, besides those usually enumerated, is wanting to account for the motion of the blood." (P. 51.)

"The inadequacy of the propulsive power of the heart and vascular tubes for the accomplishment of the circulation would seem to have been silently or indirectly felt. The old writers called in the assistance of a *vis a tergo* of the blood, the pressure of surrounding or neighbouring parts, and stated, that the vessels were so situated as to be mechanically exposed to the action of muscles, that the veins were intentionally situated close to the large arteries, to secure the advantage of the pulsation; and attributed much to the forces of gravity and capillary attraction. These formed chiefly the Harveian school; but another set of writers most ingeniously applied, in addition, the reasoning of mathematics and the experiments of physical philosophy, in the physiology of the circulation. Borelli, Keil, Hales, and some others, were the ornaments of this school: but, even with their additions, the propulsive powers, as yet allowed, seemed defective to another class of writers, who manifested equal ingenuity in their search for still other powers. The mechanism of the heart was minutely examined, and held up as a model of a forcing and suction pump. To finish this motive system of machinery, the pressure of the lungs, filled with air, on the heart, and the pressure of the atmosphere on the body, were lugged in, to eke out what was seen to be defective. This very act was a tacit acknowledgment, on the part of these mechanical theorists, that the powers of the heart were inadequate to propel the blood through its whole course. Wilson, Carson, and more lately Barry, were the able supporters of this, the sanguineous-mechanical school, which never boasted of many disciples; and its inadequacy has been, I may even say, universally admitted." (P. 52.)

"In leeches, earth-worms, zoophytes, and vegetables, there is no heart. The circulation of the blood in the lower grades of animation is effected simply by the blood and vascular tubes; and it is known that the motion of the circulating fluids, in these tubes, whether blood or sap, is carried on sometimes with an astonishing velocity and force. In every animal the absorbent and lymphatic systems have no immediate prime propelling power; but the circulation of these fluids is carried on with perfection; and phenomena sometimes shew motions rapid and forcible. The deductions to be drawn from these facts are clear and obvious. In animal hydraulics, a first-rate propelling power (as we must consider the heart to be,) is not indispensable for the circulation of its fluids, which can move and circulate without a heart.

"Even in the human subject, cases occasionally occur in which the heart is found to be wanting, and that too at a period of life when its services can least be dispensed with. Monstrous human fœtuses are recorded to have been born, plump and fat, without a heart. Every organ is formed with powers to answer some necessary purpose, as well as for the due performance of its functions: but it cannot be supposed, for it would be contrary to the laws of the animal economy, that the heart of the mother should be suddenly (or in a short space of time,) endowed with extraordinary, and what must, after the birth of the child, be superfluous, organization and powers, in order to support two circulations, her own and that of her fœtus, a *lusus naturæ*, which in her case may never be sported again. The circulation in the fœtus without a heart must be an act of the blood and vascular tubes; for very many circumstances conspire to prove the maternal heart incapable of effecting it. The extremely tortuous and unnecessarily lengthened course of the vessels, particularly those of the umbilical chord, are unfavorable for the promotion of any velocity or force of motion, as given by a first-rate impelling power. The natural and morbid instances of the absence of the heart demonstrate that it is an organ not indispensable for sanguification, and that the blood's motion can be supported, and its circulation maintained, without a heart, or any first-rate propelling power." (P. 55.)

"The blood, then, even of the arteries, possesses a power of mobility, and the circulation in this order of vessels is effected by the combined action of the heart, arteries, and blood.

"The general admission among physiologists is, that the blood moves in the capillaries unaided by the heart; and many phenomena strongly support this opinion, which, on account of the brevity demanded here, cannot be fully considered, since it is not generally disputed. The circulation, then, in this system of vessels, must depend on the blood and capillaries: and it now remains to examine if the blood has any share in propelling itself, or if it be propelled solely by the moving powers of the minute vessels." (P. 67.)

"The minute vessels have little, if any, elastic power, and the action of contractility must be the sole agent by which the vessels assist the circulation; but this assistance is a force altogether inadequate to the perfect propulsion of the capillary blood. Daily observation will readily present physiological facts in confirmation, and will shew the capillaries of some membranes in a non-impelling state, and therefore not moving the blood. That these small vessels cannot be contracting at the same time that they are dilated, is a truism too self-evident to fear contradiction; and an increased quantity of blood must dilate them. Such an increased quantity accumulates during the natural act of blushing, which, in some cases, continues for a great length of time; but when the cavity of the vessel is so surcharged, and its sides so dilated, the circulation still proceeds with even an increased velocity. The circulation, then, must be effected, and continued, by the moving power of the blood; for there is no other power, physical, mechanical, or vital, to which it can, by any possibility, be attributed. The vessels, it is to be recollected, are dilated, and contraction is the force which, by pressing on the blood, moves and circulates that fluid. In this dilated state the blood and vessels remain, till the contractile power of the middle coat reacts, and, by diminishing the cavity of the canal, restores it to its usual bore. Pathology abounds with such facts; for inflammation presents such a state of incapacitated capillaries, in every membrane and organ of the body. The weight of a column of arterial blood does not furnish sufficient force; for, from the position of the vessels, gravity does not affect it; but the blood is regularly transmitted. Capillary attraction, of whose laws natural philosophy is unfortunately very ignorant, cannot account for this; for the diameter of many of the larger capillaries is too great to permit the action of this kind of attraction. Indeed, it is very doubtful if it operates in any degree, even in the very smallest of these vessels, and an immense number of them are exceedingly minute. The working of animal machinery, and its many oily surfaces, do not favor the operations of this power; but still, if I were requested to select any part of the body in proof of the self-motion of the blood, I would triumphantly point to the capillary system. These small, but very important, vessels, are as often out of a condition for assisting the circulatory motion, as they are in a state favorable for the transmission of blood; but the blood, assisted or not, moves on by its own moving force.

"The current of the blood is observed often to be increased in velocity, sometimes in quantity, when passing from the capillaries to the veins, which is owing to the conjoint action of the blood and the small vessels. The capillaries are extremely numerous, amounting to myriads, and the vast multitude of them extremely small, descending to $\frac{1}{200}$ and $\frac{1}{2000}$ part of an inch. Some are even stated by writers to be much more minute. The force of such vessels must be small, and the capillary *vis a tergo* of the blood flowing from such diminutive passages trifling; particularly when the physiology of the muscular texture, and the physical force of friction, are recollected. The retardations will be inversely as the diameter of the tubes, and directly as the viscidity of the blood. From these data, it is a fair inference that the motion of the venous blood must depend on the blood and veins; for it can bring no acquired force from the capillaries.

"The veins can render but little assistance to the circulation. Their structure forbids it; and their coats are, in general, possessed of very little contractile power. The irregularity in the distribution of a middle coat, its thinness, and total absence in a great number of these vessels, and even in parts of the same vessel, show that its presence is not indispensable for the motion of the blood. The venous circulation, then, is effected and perfected by the power of some other matter; and as there is no other than the blood, the circulating force must therefore be some moving power of the blood. The sinuses of the brain, being the venous receptacles into which the innumerable small venous branches of this divine organ pour their tributary streams, are formed only of the common inner lining of all the vessels, and the fibrous membrane of the *dura mater*, which are textures possessing no contractile powers. In spite, however, of this want of assistance from the vessels, the full and easy current of the circulation is regularly maintained, and the blood is poured into the veins external to the encephalon, by means of its own moving powers.

" When we consider that the greatest quantity of venous blood moves against the laws of gravity, and all the known laws of dead fluids, in vessels perfectly flaccid, and confessedly too deficient in contractile power, we cannot withhold our confession of the truth, that the blood must have a power of motion within itself. When we see veins so distended that they must have their middle coat placed beyond its sphere of contractile action, as in varicocele, where the blood is moving in vessels which only serve to confine its fluid; when we see the great tortuosity and plexuses of venous trunks; when we see their frequent and innumerable branchings and joinings, so as to form so many ovals, we must admit that the solid textures do not effectually propel the blood; and, knowing that all the actions of the vascular system are accomplished by the powers of its own matter, we must admit that the blood has motion inherent in its structure. We see it placed in situations where it is deprived of every adequate means of containing circulation, except by the force or exercise of its own moving powers.

" Nor is it surprising that the blood should move itself; it is only accordant with the phenomena of animated nature. The great characteristic between organic and inorganic matter is that the former possesses within itself a power as well of producing as of stopping motion in its own body; while in the latter a body must receive its motion from the chemical and mechanical impression of some other body, and, when in action, must be stopped by another power. If a stone, previously at rest, be dropped from some elevation, it will continue in motion by the laws of gravitation, throughout all space, until it is stayed in its progress by some greater power, as the earth. A locomotive engine, put in motion by the expansive force of steam, will continue moving as long as there is any steam, unless prevented by some other machinery or power. But neither the stone nor the engine can of itself either begin or stop its motions. On the contrary, well-organized structures, fluid and solid, can of themselves both move and stop.

"Even vital fluid has, therefore, a power of self-motion; and this supposition will furnish the only rational mode of accounting for the currents of the chyle and vegetable sap. Motion, in these last two systems, animal absorbent and vegetable sap-vessels, is adequately accomplished without a first-rate propelling power. The membranes forming the vessels themselves are obviously too weak and delicate to possess much propulsive force; but physiologists have evaded all inquiries on the subject, by attributing this power to absorption, which I consider to be an expression used as a cloak for ignorance. Some of them, perceiving that this property did not afford a sufficient explanation for the progressive motion and selection of these fluid bodies, called to their aid a *vita propria*, which, in fact, explains nothing, and only makes 'darkness visible.'" (P. 68.)

We are far from being affected with the Nosologimania which the Cullenians, and those emanating from that school, were famed for; still we think the following sweeping condemnation scarcely just: "The present complicated nosologies are, therefore, not only perfectly useless, but injurious, since the cause of the pathological states of structure is clearly attributable to one variety or other of inflammation;" and we acknowledge our obtuseness in not perceiving the vast superiority of the arrangement here proposed.

"Medical philosophers and nosologists have attempted to classify fevers, and, as might be expected, all their labours have proved abortive; for they collected groups of symptoms, and dignified them with the title of fever. If distinctions are to be made in fevers, (which I regard as absolutely unnecessary,) I would designate them by the predominance of the disordered state and actions of one of the principal systems. The digestive system is either more or less disordered in every case; but in some instances the greatest disorder obviously prevails in it, and the fever might thus be called the gastric. In other cases, the actions belonging to the nervous or vascular systems may predominate, the symptoms belonging to one or the other being most strongly marked, and the fever might be called the nervous or vascular accordingly. The reason of the disorder of one system exceeding the others, will not unusually be found in the greater inflammation of that part of it which enters into the affected texture: for instance, if the capillaries of the affected part be more inflamed than either the absorbents or nerves, the associated disorder of the vascular system is then likely to exceed that of the digestive or nervous. The state of the organization of those organs which are the centres of their respective systems will influence the fever; but, in whatever system the disorder may be greatest at first, it is liable, during the progress of the disease, to prevail in the highest degree in another, and, towards the close, to be equal in all. In many cases, however, it is impossible to ascertain that the fever of any one system is greater than that of another; for all the systems are alike disordered." (P. 211.)

We are happy to find, however, that, although we are far more old-fashioned in our theories of disease, that we should not be far behind our author in its treatment, and that our practice would not be opposed in many respects; the system of counter-irritation and incisions in erysipelas being the most important points of difference.

"Cupping, and incisions, are other means practised for the attainment of the same objects as leeches. Cupping is beneficially applied on the skin, over deep-seated parts which are chronically inflamed; and, in addition to the removal of blood, accumulates a quantity of capillary blood in the cupped textures, which very rarely fall into inflammation. Incisions have lately been made, by several distinguished surgeons, on parts labouring under compound erysipelas: one surgeon recommends the incisions to measure an inch, or an inch and a half, to go deep, and to number from six to eighteen: a second, more bold, will have the scalpel to make few incisions, but these enormous ones, extending over all the erysipelatous surface; one cut on this plan has even measured eighteen inches, so that two or three long cuts generally suffice: and a third will have the other surface studded over with numerous small apertures from the pricks of a lancet. If the principles of this treatment be examined, they will be found applicable to all inflammations where tension, and accumulation of fluids, take place; and I have therefore cut parts in a state of phlegmon, with the same effect as in compound erysipelas. But does this cutting practice offer any superiority over leeches, sufficient to counterbalance its severity, the great, if not occasionally even fatal, hazard from hemorrhage, and the certain fact that perfect resolution can never be obtained; for after the abatement of inflammation, there will still remain numerous small, or a few immense, gaping wounds, or a multitude of little pricks or ulcers to be healed, which is a consideration of no minor importance in bad constitutions. Incisions, by dividing the vessels, allow a free flow of blood, and the escape of the effused fluids, which leeches do not, though they unburden the vessels, by removing the blood; so that the same object is attained by both means, with this difference, that leeches remove not the effused fluids, but leave no wounds, which incisions do. The former are, therefore, generally preferable to the latter, since the effusions can be, and often are, readily absorbed." (P. 266.)

"There is another class of local remedies in general use, called counter-irritants, blisters, cauteries, escars, mustard poultices, ointments of tartarized antimony, and such like. The modus operandi of these remedies has been the subject of a variety of conjectures: some have looked to anatomy for a solution of the enigma, and have endeavoured to trace the route of some branch of a blood-vessel from the inflamed to the blistered part; others have attributed it to nervous agency; and others to sympathy. I believe the true solution will be found in the circumstance of another and new malady being produced by them, affecting some structure, or its organizing machinery, and the constitution. It is, in fact, an attempt to cure one disease by making another, either in the same structure, or on some other. These stimuli are applied on the inflamed structure itself; on healthy parts situated near the disordered; on another texture of the same anatomical nature, or otherwise; or on parts very remote." (P. 269.)

"On the other hand, if it be attempted by these stimulants to transfer the inflammation of an important part to an unimportant one, the attempt, in the vast majority of cases, will fail; so that there will be two diseases, or two parts affected, instead of one. Even when by chance it does succeed, the original malady is very liable to return, and create a new one, which is another source of disorder and of fever. These remedies are sometimes applied to parts very remote from the original malady, as sinapisms to the feet for affections of the head, but for what purpose I have not been able to discover. This principle, or practice, is a remnant of the antiquated doctrines of derivation, and is not worthy of any serious attention. In short, the only utility of this class of stimulants is to give pain; and when pain becomes desirable, or a certain benefit, or a source of pleasure, these remedies may be used; but, as long as pain is an evil, let them be discarded. They are unnecessary, and very frequently injurious." (P. 271.)

There are many other points on which we differ considerably from our author, besides those to which we have above adverted; such as his explanation of the cause of the buffy coat of the blood, for we cannot conceive that it arises from forcible and *rapid* coagulation:" but we have neither time nor space for further observations, and therefore, in conclusion, can only add, that, although we have read this treatise with much attention, we are still far from being convinced "that the beginning of every morbid evil is a species of inflammation;" because, if it be so admitted, the received idea of inflammation must be changed for a definition essentially different; and we are not persuaded that any advantages would result from the change, equivalent to the distraction it would cause.

*On Hybernation.* By MARSHALL HALL, M.D., F.R.S. L. & E., &c. (From the *Philosophical Transactions.*)

IN this memoir, the author follows out the principle developed in the essay noticed in a previous number, entitled a "Theory of the Inverse Ratio which subsists between the Respiration and Irritability, in the Animal Kingdom." Some of the results obtained are curious and interesting. We shall condense a part of the experimental inquiry.

"That peculiar condition of certain mammalia during the winter season, which has been designated hybernation, has been aptly compared by various authors to ordinary sleep. In both the respiration is diminished. This fact was first determined, in regard to sleep, by Messrs. Allen and Pepys. It obtains in a much higher degree in the state of hybernation. It is highly probable that in sleep, as in hybernation, the irritability of the muscular fibre becomes augmented. These two conditions of the animal system may therefore mutually illustrate each other.

"Ordinary sleep is similar to the sleep of the hybernating animal; and the sleep of the hybernating animal is similar to that deeper sleep, or lethargy, which is designated hybernation. We are thus led to trace a connexion between the recurrent sleep of all animals, and the deep and protracted sleep of a few.

"1. *Of the Sleep of hybernating Animals.* In the sleep of the hybernating animal, the respiration is more or less impaired: if the animal be placed in circumstances which best admit of observation, the acts of respiration will be found to have greatly diminished; if it be placed in the pneumatometer, little alteration is induced in the bulk of the air; if its temperature be taken by the thermometer, it will be found to be many degrees lower than that of the animal in its active state; if it be deprived of atmospheric air, it is not immediately incommoded or injured." (P. 1.)

"The respiration is very nearly suspended in hybernation. That this function almost ceases, is proved, 1st, by the absence of all detectible respiratory acts; 2dly, by the almost entire absence of any change in the air of the pueumatometer; 3dly, by the subsidence of the temperature to that of the atmosphere; and 4thly, by the capability of supporting, for a great length of time, the entire privation of air." (P. 4.)

The following may serve as an example of the experiments whence the conclusions in this paper have been drawn, and some of which are, in the original memoir, reduced to a tabular form, and the registers kept for many days successively.

"On January the 28th, the temperature of the atmosphere being 42°, I placed a bat in the most perfect state of hybernation, and undisturbed quiet, in the pneumatometer, during the whole night, a space of ten hours, from 1h. 30m. to 11h. 30m. There was no perceptible absorption of gas.

"Having roused the animal a little, I replaced it in the pneumatometer, and continued to disturb it from time to time, by moving the apparatus. It continued inactive, and between the hours of 1h. 20m. and 4h., there was the absorption of one cubic inch only of gas.

"Being much roused at four o'clock, and replaced in the pneumatometer, the bat now continued moving about incessantly; in one hour, five cubic inches had disappeared. It was then removed. A further absorption took place of .8 of a cubic inch of gas.

"Thus the same little animal, which, in a state of hybernation, passed ten hours without respiration, absorbed or converted 5.8 cubic inches of oxygen gas into carbonic acid, in one hour, when in a state of activity. In an intermediate condition, it removed one cubic inch of oxygen in two hours and forty minutes.

"I repeated this experiment on February the 18th. A bat, in a state of perfect hybernation, was placed in the pneumatometer, and remained in it during the space of twenty-four hours. There was now the indication of a very slight absorption of gas, not, however, amounting to a cubic inch.

"On February the 22d, I repeated this experiment once more, continuing it during the space of sixty hours; the thermometer descended gradually, but irregularly, from 41° to 38°; the result is given in the subjoined Table.

| Date, | | External Temp. | Absorption. | Duration. |
|---|---|---|---|---|
| February 22 | 11 P.M. | 41° | | |
| 23 | 11 A.M. | 38½ | ·8 | 12h. |
| | 11 P.M. | 39½ | ·75 | 12 |
| 24 | 11 A.M. | 38 | ·5 | 14 |
| | 11 P.M. | 39 | ·75 | 12 |
| 25 | 11 A.M. | 38 | ·6 | 12 |
| | | | 3·4 | 60 |

"From this experiment it appears that 3.4 cubic inches of oxygen gas disappeared in sixty hours, from the respiration of a bat in the state of lethargy. It has been seen that in a state of activity, an equal quantity of this gas disappeared in less than half that number of minutes. The respiration of the hybernating bat descends to a sub-reptile state; it will be seen shortly that the irritability of the heart, and of the muscular fibre generally, is proportionably augmented.

"In this experiment it is probable that the lethargy of the animal was not quite complete." (P. 5.)

Another lengthened series of observations was made to show that "the temperature of the hybernating animal accurately follows that of the atmosphere;" but for the detail of these we must refer the reader to the original memoir: the results are as follow:

"When the changes of temperature in the latter are slight, the two thermometers denote the same temperature. If these changes are greater and more rapid, the temperature of the animal is a little lower or higher, according as the external temperature rises or falls; a little time being obviously required for the animal to attain that temperature.

"Similar observations were made during the first three days of February. On the 4th, however, the temperature of the atmosphere rose to 50½°; that of the animal was now 82°, and there was a considerable restlessness. On the 6th, the temperature of the atmosphere had fallen to 47½°, and that of the animal to 48°, whilst there was a return of the lethargy.

"After this period there were the same equal alterations of temperature in the animal and in the atmosphere, observed in the month of January.

"It is only necessary to add to these observations, that the internal temperature is about three degrees higher than that of the epigastrium. In two bats, the external temperature of each of which was 36°, a fine thermometer, with an extremely minute cylindrical bulb, passed gently into the stomach, rose to 39°." (P. 8.)

After quoting some experiments of Jenner, Edwards, and Spallanzani, our author continues: "It is in strict accordance with these facts that the lethargic animal is enabled to bear the total abstraction of atmospheric air, or oxygen gas, for a considerable period of time."

"A bat which was lethargic in an atmosphere of 36° was immersed in water of 41°. It moved about a little, and expelled bubbles of air from its lungs. It was kept in the water during sixteen minutes, and then removed. It appeared to be uninjured by the experiment.

"A hedgehog, which had been so lethargic in an atmosphere of 40° as not to awake for food during several days, was immersed in water of 42°. It moved about and expelled air from its lungs. It was retained under the water during 22½ minutes: it was then removed. It appeared uninjured.

"It seems probable that the motions observed in these animals were excited through the medium of the cutaneous nerves.

"The power of supporting the abstraction of oxygen gas, or atmospheric air, belongs solely to the hybernating state, and is no property of the hybernating animal in its state of activity. After having found that the dormant bat, in summer, supported immersion in water, during eleven minutes, uninjured, I was anxious to know whether the active hedgehog possessed the same power, I immersed one of these animals in water. It expired in three minutes, the period in which immersion proves fatal to the other mammalia. Sir Anthony Carlisle has, therefore, committed an error, somewhat similar to that of Dr. Edwards, when he asserts that 'animals of the class mammalia, which hybernate and become torpid in winter, have at all times a power of subsisting under a confined respiration, which would destroy other animals not having this peculiar habit.' The power of bearing a suspended respiration is an induced state. It depends upon sleep or lethargy themselves, and their effect in impairing or suspending respiration; and upon the peculiar power of the left side of the heart, of becoming veno-contractile under these circumstances.

"2. *Of the Irritability*. The single fact of a power of sustaining the privation of air, without loss of life, leads alone to the inference that the irritability is greatly augmented in the state of hybernation. This inference flows from the law so fully stated in my former paper, and the fact is one of its most remarkable illustrations and confirmations.

"It might have been inferred from these premises, that the beat of the heart would continue longer after decapitation in the state of hybernation than in the state of activity in the same animal: an inference at once most singular and correct.

"This view receives the fullest confirmation from the following remarkable experiment: On March the 9th, soon after midnight, I took a hedgehog which had been in a state of uninterrupted lethargy during 150 hours, and divided the spinal marrow just below the occiput; I then removed the brain and destroyed the whole spinal marrow as gently as possible. The action of the heart continued vigorous during four hours, when, seeing no prospect of a termination to the experiment, I resolved to envelop the animal in a wet cloth, and leave it until early in the morning. At seven o'clock A.M. the beat of both sides of the heart still continued. They still continued to move at ten A.M., each auricle and each ventricle contracting quite distinctly. At half after eleven A.M. all were equally motionless; yet all equally contracted on being stimulated by the point of a penknife. At noon the two ventricles were alike unmoved on being irritated as before; but both auricles contracted. Both auricles and ventricles were shortly afterwards inirritable.

"This experiment is the most extraordinary of those which have been performed upon the mammalia. It proves several interesting and important points: 1. That the irritability of the heart is augmented in continued lethargy in an extraordinary degree. 2. That the irritability of the left side of the heart is then little, if at all, less irritable than the right, that it is, in fact, veno-contractile. 3. That, in this condition of the animal system, the action of the heart continues for a considerable period independently of the brain and spinal marrow.

"On April the 20th, at six o'clock in the evening, the temperature of the atmosphere being 53°, a comparative experiment was made upon a hedgehog in its state of activity: the spinal marrow was simply divided at the occiput: the beat of the right ventricle continued upwards of two hours, that of the left ventricle ceased almost immediately; the left auricle ceased to beat in less than a quarter of an hour; the right auricle also ceased to beat long before the right ventricle." (P. 11.)

We need make no comments on the above experiments; and the following observations are likewise worthy attention, as being the only really satisfactory ones which have been made on the circulation of hybernating animals.

"The wing of the bat affords an admirable opportunity of observing the condition of the circulation during hybernation. But it requires peculiar management. If the animal be taken from its cage, and the wing extended under the miscroscope, it is roused by the operation, and its respiratory and other movements are so excited, that all accurate observation of the condition of the circulation in the minute vessels is completely frustrated. Still greater caution is required in this case, than even in the observation of the respiration and temperature.

"After some fruitless trials, I at length succeeded perfectly in obtaining a view of the minute circulation undisturbed. Having placed the animal, in its state of hybernation, in a little box of mahogany, I gently drew out its wing through a crevice made in the side of the box; I fixed the tip of the extended wing between portions of cork; I then attached the box and the cork to a piece of plate-glass; and, lastly, I left the animal in this situation, in a cold atmosphere, to resume its lethargy.

"I could now quietly convey the animal ready prepared, and place it in the field of the miscroscope without disturbing its slumbers, and observe the condition of the circulation.

"In this manner I have ascertained that, although the respiration be suspended, the circulation continues uninterruptedly. It is slow in the minute arteries and veins; the beat of the heart is regular, and generally about twenty-eight times in the minute.

"We might be disposed to view the condition of the circulation in the state of hybernation as being reptile, or analogous to that of the batrachian tribes. But when we reflect that the respiration is nearly, if not totally, suspended, and that the blood is venous, we must view the condition of the circulation as in a lower condition still, and, as it were, sub-reptile. It may, indeed, be rather compared to that state of the circulation which is observed in the frog from which the brain and spinal marrow have been removed by minute portions at distant intervals.

"In fact, in the midst of a suspended respiration, and an impaired condition of some other functions, one vital property is augmented. This is the irritability, and especially the irritability of the left side of the heart. The left side of the heart, which is, in the hybernating animal, in its state of activity, as in all the other mammalia, only arterio-contractile, becomes veno-contractile.

"This phenomenon is one of the most remarkable presented to me in the whole animal kingdom. It forms the single exception to the most general rule, amongst animals which possess a double heart. It accounts for the possibility of immersion in water or a noxious gas, without drowning or asphyxia; and it accounts for the possibility of a suspended respiration, without the feeling of oppression or pain, although sensation be unimpaired. It is, in a word, this peculiar phenomenon, which, conjoined with the peculiar effect of sleep in inducing diminished respiration in hybernating animals, constitutes the susceptibility and capability of taking on the hybernating state. On the other hand, as the rapid circulation of a highly arterialized blood in the brain and spinal marrow of birds probably conduces to their activity, the slow circulation of a venous blood, doubtless contributes to the lethargy of the hybernating animal." (P. 17.)

The gradations which have been observed in the profoundness of the lethargy, and in the period of its duration, have, in each stage, especial reference to the habits and wants of the animal.

"There is much difference in the powers of digestion, and in the fact of omitting to take food, in the hybernation of different animals. The bat, being insectivorous, would awake in vain; no food could be found: the hedgehog might obtain snails or worms, if the ground were not very hard from frost; the dormouse would find less difficulty in meeting with grain and fruits. We accordingly observe a remarkable difference in the habits of awaking from their lethargy or hybernation, in these different animals.

"I have observed no disposition to awake at all in the bat, except from external warmth or excitement. If the temperature be about 40° or 45°, the hedgehog, on the other hand, awakes, after various intervals of two, three, or four days passed in lethargy, to take food; and again returns to its state of hybernation. The dormouse, under similar circumstances, awakes daily.

"Proportionate to the disposition to awake and take food, is the state of the functions of the stomach, bowels, and kidneys. The dormouse and the hedgehog pass the fæces and urine in abundance during their intervals of activity. The bat is scarcely observed to have any excretions during its continued lethargy.

"In the dormouse and the hedgehog, the sense of hunger appears to rouse the animal from its hybernation, whilst the food taken conduces to a return of the state of lethargy. It has already been observed, that there are alternations between activity and lethargy in this animal, with the taking of food, in temperatures about 40° or 45°. Nevertheless, abstinence doubtless conduces to hybernation, by rendering the system more susceptible of the influence of cold, in inducing sleep and the loss of temperature. The hedgehog, which awakes from its hybernation, and does not eat, returns to its lethargy sooner than the one which is allowed food." (P. 19.)

We reluctantly pass by the sections relating to "Torpor from Cold," "Sensibility," "Muscular Motility and Reviviscence," without transferring any of the extracts we had marked to our pages; but space forbids us to do more than quote the author's recapitulation.

"The object of this paper has been to treat of the singular phenomena of hybernation, and especially to point out the remarkable application of the law stated in my former paper, to the active and lethargic states of the hybernating animal.

"1. The natural sleep of the hybernating animal differs greatly, yet only in degree, from the sleep of any other animal.

"2. This sleep passes insensibly into the state of true hybernation, which is more profound, as the blood loses its arterial character; for,

"3. In hybernation, the respiration and the evolution of heat are nearly suspended.

"4. The irritability is, at the same time, singularly augmented; and the animal bears proportionately the privation of air.

"5. The nervous sensibility and the muscular motility are unimpaired.

"6. There is the singular phenomenon of this unimpaired sensibility, and the capability of bearing the privation of air without pain; a fact which receives an interesting and perfect explanation from the additional fact of the augmented irritability or veno-contractibility of the left side of the heart.

"7. There is an important distinction between true hybernation and torpor from cold, not attended to by physiologists.

"8. Severe cold, like all other causes of pain, rouses the hybernating animal from its lethargy; and, if continued, induces the state of torpor.

"In conclusion, one of the most general effects of sleep, is to impair the respiration, and, with that function, the evolution of animal temperature. The impaired state of the respiration induces a less arterial condition of the blood, which then becomes unfit for stimulating the heart; accumulation of the blood takes place in the pulmonary veins and left auricle; a sense of oppression is induced, and the animal is either roused to draw a deep sigh, or awakes altogether.

"Such are the phenomena in animals in which the heart has not the faculty of taking on an augmented state of irritability, with this lessened degree of stimulus. But in those animals which do possess this faculty, a property which constitutes the power of hybernation, the heart continues the circulation of the blood, more slowly indeed, but not less perfectly, although its arterial character be diminished and its stimulant property impaired. No repletion of the pulmonary veins and of the left auricle, no sense of oppression, is induced, and the animal is not roused; the respiration continues low, the temperature falls, and the animal can bear, for a short period, the abstraction of atmospheric air.

"All the phenomena of hybernation originate, then, in the susceptibility of augmented irritability. The state of sleep, which may be viewed as the first stage of hybernation, induces an impaired degree of respiration. This would soon be attended with pain, if the irritability of the heart were not at the same time augmented, so as to carry on the circulation of a less arterial blood, and the animal would draw a deep sigh, would augment its respiration, or awake. Occasional sighs are, indeed, observed in the sleep of all animals, except the hybernating. In these, the circulation goes on uninterruptedly, with a diminished respiration, by the means of an augmented irritability. There is no stagnation of the blood at the heart; consequently, no uneasiness; and the animal becomes more and more lethargic, as the circulation of a venous blood is more complete. This lethargy is eventually interrupted by circumstances which break ordinary sleep, as external stimuli, or the calls of appetite.

"Moderate cold disposes to sleep, to lethargy; but severer cold induces a different condition of the system, that of torpor. Sleep is the medium between such moderate cold and the phenomena of hybernation; torpor is the immediate effect of the severer degrees of cold.

"This investigation naturally leads to that of the comparative conditions of the respiration and of the irritability, in the pupa and perfect states of some species of the insect tribes. There is much reason to suppose that these states are respectively similar to those of lethargy and activity in the hybernating animal." (P. 24.)

A peroration is not needed to express our opinion of this paper; the extracts we have made demonstrate our belief that it is a very valuable contribution to modern physiology, and that the experiments and theories have not only truth but novelty to recommend them.

